# Supplementary material for: How to achieve a higher selection plateau in forest tree breeding? Fostering heterozygote × homozygote relationships in optimal contribution selection in the case study of Populus nigra
Source: Evol Appl. 2021 Sep 21;14(11):2635–46. doi: 10.1111/eva.13300 (PMC8591327; doi:10.1111/eva.13300)
Supplement: Supplementary file 1 — Supplementary Material [file EVA-14-2635-s001.docx]

Supplementary Material

1. **Proof of equation (2)**

Let X be the matrix describing the genotypes in the population, with L rows (number of markers) and N columns (number of individuals). The two homozygous states are encoded as −1 and 1, and the heterozygous state as 0 (as in Van Raden, 2008). Let then Q defined as follows:

Q = 0.5 L_NxN_ − 2 (|X| − ½_LxN_)^T^ (|X| − ½_LxN_),

where L_NxN_ a matrix of size N x N with each value equal to L, |X| being the matrix X with each value transformed to its absolute value, and ½_LxN_ a matrix of size L x N with each value equal to ½. This matrix Q is a matrix of size N x N, for which each value is the count of 0 x 1 or 0 x -1. Likewise, let W defined as follows:

W = ( (X − 1_LxN_) ၀ (X + 1_LxN_) )^T^ ( (X − 1_LxN_) ၀ (X + 1_LxN_) ),

with identical previous notation, and ၀ the Hadamard product. This matrix W is a matrix of size N x N, for which each value is the count of 0 x 0. Let now *c* be the genetic contribution, a vector of size N, for which each value is between 0 and 0.5 and for which the sum is equal to 1. Therefore, we have the following:

c^T^Qc = 0.5 L − 2 c^T^ (|X| − ½_LxN_)^T^ (|X| − ½_LxN_) c

= 0.5 L − 2 ∑_k_ ( ∑_i_ c_i_ (|x_k,i_| − ½) )^2^ = 0.5 L − 2 ∑_k_ (ho_k_ − ½)^2^, with ho_k_ the total contribution of homozygous individuals for the locus k,

= 0.5 L − 2 ∑_k_ (ho_k_^2^ − ho_k_ + ¼) = 2 ∑k ho_k_ (1 − ho_k_) = 2 Ho^T^ He,

where Ho is the vector of ho_k_, and He the equivalent for heterozygous individuals.

As for W, using similar reasoning and remarking that c ( (X − 1_LxN_) ၀ (X + 1_LxN_) ) is equal to He, we can deduce that :

c^T^Wc = He^T^ He.

Therefore, we can state that:

c^T^G*c = c^T^Gc + β Ho^T^He + γ He^T^He.

Literature cited:

VanRaden, P.M. (2008). Efficient methods to compute genomic predictions. Journal of dairy science. 91(11), 4414-4423. doi: 10.3168/jds.2007-0980

1. **Why OCS cannot be formulated explicitly with mating information**

Consider a breeding program in which we want to implement an Optimal Contribution Selection (OCS) on a population of N individuals, for an offspring population size of N_off_, with L biallelic loci. The ideal situation would be to optimize genetic gain and coancestry based on a genetic contribution for each mating, recorded in a matrix **C** (size NxN), instead of a vector **c** and allocating mating in a second step. Without loss of generality, for this demonstration, the genetic contribution are recorded as the *number* of offspring, thus summing to 2N_off_, and not 1. The genetic gain would be formulated as E(Y_t+1_) = Y^T^C.**1**, where Y is the vector (size Nx1) of parent breeding values and **1** a vector of 1 (size Nx1). The expected coancestry is E(**1**^T^G_t+1_**1**), or in other words, the sum of all terms of the expected matrix E(G_t+1_), where G_t+1_ = X_t+1_^T^X_t+1_, with X_t+1_ the matrix of the genotypes at generation t+1 (size LxN_off_) encoded with -1/0/1 (see the main text). Every element of E(G_t+1_) can easily be deduced, as the expected homozygosity (diagonal elements of G_t+1_) of an offspring from the mating of the parents i and j (or i x j) is equal to ½ (g_ij_ + L), where g_ij_ is the relatedness of the parents (the *i*-th row and *j*-th column of the matrix G). Likewise, the expected relatedness (off-diagonal elements of G_t+1_) between an offspring from the mating i x j and an offspring from the mating k x h, is ¼ (g_ik_ + g_ih_ + g_jk_ + g_jh_). With these formula, it is possible to deduce E(G_t+1_) with the matrix M (size N_off_ x N), twice the matrix defined in Quaas (1988), where the *i-*th line indicates the two parents of the *i*-th offspring (*e.g.*, if the *i*-th offspring is from the *j*-th and *k*-th parents, on the *i*-th line of M, the *j*-th and *k*-th columns will be equal to 1; otherwise equal to 0). The matrix M carries the same information than **C**, and it is easy to transform one to the other. The formula of E(G_t+1_) is as follows:

E(G_t+1_) = ¼ (MGM^T^ – diag(MD – 2**L**)),

where M is the matrix of mating, G the parent genomic relationship matrix defined as X^T^X (X is the matrix of the genotypes, size LxN, encoded with -1/0/1), diag(.) is the operator that transforms a vector into a diagonal matrix, D the vector of size Nx1 composed of the diagonal elements of G, and **L** a vector of size N_off_x1 filled with the value L. The second term is a correction term for the diagonal element of the first term, coming from the fact that the formula of the diagonal and off-diagonal elements of E(G_t+1_) are algebraically different.

In the ideal case, we would be able to optimize the objective function (1 – α)E(Y_t+1_) - α E(**1**^T^G_t+1_**1**) for a given α (with some design constraint) to find the best M*. However:

E(**1**^T^G_t+1_**1**) = **1**^T^E(G_t+1_)**1**

= ¼ ( **1**^T^MGM^T^ **1**  – **1**^T^diag(MD – 2**L**)**1** )

= ¼ (**c**^T^Gc + h^T^**c**),

where h is the vector of size Nx1 for which the *i-*th element is the heterozygosity of the *i*-th individual (defined as L – g_ii_). In addition, E(Y_t+1_) = Y^T^**c**. As we can see, the objective function cannot distinguish M and **c**, so the mating information is completely lost by construction. We can notice an additional term proportional to heterozygosity, that we did not account for in the main text.

This formula demonstrates that OCS cannot integrate the mating information if the objective function is formulated with the expected values of breeding values and coancestries. Mating information impact the variance at generation t+1, or the expected values at t+2; therefore, it would be possible to optimize over mating if and only if such quantities were added in the objective function.

Literature cited:

Quaas, R. L. (1988). Additive genetic model with groups and relationships. *Journal of Dairy Science*, *71*(5), 1338-1345. [doi:10.3168/jds.S0022-0302(88)79691-5](https://doi.org/10.3168/jds.S0022-0302(88)79691-5)

1. **Mate allocation and linear programming**

Once the optimal genetic contributions are obtained from the resolution of the quadratic programming defined in the main text, we can either apply random mating, or we can optimize mating. In this paper, we proposed to optimize mating over the expected homozygosity, or tr(E(G_t+1_)), where tr(.) is the trace, and E(G_t+1_) is as defined above. Using the notation of the previous section, we can reformulate tr(E(G_t+1_)) as a linear function of ∑g_ij_**C_ij_**, where g_ij_ are elements of G*, and **C_ij_** elements of **C**. We used G* instead of G to stay inline with the OCS process, performed with G*. The trace can alternatively be formulated as the product of the vectors S and T (size 1x(N(N-1)/2)), composed of the upper triangular elements (without the diagonal elements) of G* and **C**, respectively. We do not account for the diagonal elements, because we assume no selfing. Let A be a permutation matrix (size Nx(N(N-1)/2) ), so that C = A.S. Since T^T^S is a linear function of tr(E(G_t+1_)), the optimization problem over S can be formulated as:

min. T^T^S

s.t. A.S = **c**, and for all i, 0 < S_i_ < 1,

where the first constraint translates the conservation of the genetic contributions over the optimization process, and the second translates an operational constraint about the boundaries of the elements of S. The optimal S is then multiplied by N_off_, then rounded so that the contributions become the actual number of offspring per mating (the sum of all discrete elements are controlled to be exactly N_off_). The result is then exactly the mating plan, that is directly used to generate the next generation. One can note that there is no stochasticity in the computation of the mating plan, so that the Mendelian sampling and environmental deviation are the only sources of stochasticity.

1. **Interplay between β and γ**

In addition to main text, we introduce here an additional parameter γ as the value for the He x He relationship (originally fixed to 0). In this section, we investigate the interplay of α, β and γ in simulations with perfect heritability (h² = 1), meaning that the genetic evaluation was ideal. Model (2) showed a much stronger effect of β on both genetic gain and coancestry than γ (Figures S4 and S5). For high values of α (α > 0.4), the proportion of genetic gain variance explained was much higher for β (> 30%) than for γ (< 5%), again suggesting a stronger effect of He x Ho pairs on genetic gain than He x He pairs (Figure S6). Indeed, the estimated values of β were increasingly negative as α increased, corroborating that favoring He x Ho relationship (negative β) improves genetic gain. On the other hand, the estimates of γ remained close to 0, showing its negligibility. Likewise, for coancestry, β had a higher explanatory power (> 70%) than γ (< 10%, Figure S7). The effects of β increased as α increased, showing that favoring He x Ho relationship (β < 0) would enable to maintain a lower coancestry.

In the OCS formulation, α remains the most important factor determining gain and diversity in the population dynamics over generations. In its classical formulation, i.e. β equal to zero, OCS has become one of the most efficient strategies with a combination of genetic gain and coancestry making the very edge of the Pareto optimum, as expected. When allowing for different weightings for the breeding relationship through the extra parameter β and γ, it was clear that pair choice had also a non-negligible role that increased in importance over generations, both for genetic gain and coancestry. Mendelian sampling, seen through the implicit formulation derived here, was then the second most important factor in the optimal outputs. We could safely conjecture that through an explicit formulation such importance could be further enhanced, although it would probably remain second to the effect of α.

For a given α, and notably for the highest values, β modulated the genetic gain over generations in a direct and conspicuous manner (Figure 2): promoting He x Ho relationship (i.e., β < 0) increased genetic gain, while penalizing He x Ho relationship (β > 0) decreased genetic gain. Increasing genetic gain usually means: (i) a lower genetic (and genic) variance after selection, (ii) a higher level of fixation of favorable alleles, which constitutes the matter making up genetic gain, and (iii) a higher level of negative linkage disequilibrium covariance due to the Bulmer effect.

Therefore, the extra gain obtained from β < 0 could come from using more efficiently the genic variance, resulting in more depletion compared to that of higher β levels (Figure S8), and thus converting this available variation into favorable allele fixation, or likewise unfavorable allele elimination. Compared to β = 0, β = -0.5 was always on the side of the Pareto curve with higher coancestry (and gain). Promoting He x Ho relationship, which could theoretically boost segregation in descendants more than any other combination, could be the source of the extra genetic variation that is made available favorably for selection under β < 0. On the other hand, promoting segregation across loci could be a source of linkage disequilibrium generating negative covariation of effects, as the chances of uniting alleles of opposing effects across neighboring loci could be increased and further boosted by selection. Such a tendency is clearly shown in Figure S8.

When looking at the opposite extreme, when β > 0 and notably at β = 1, genetic gain is lowest while the levels of genotypic covariance due to linkage disequilibrium are soon recovered from initial Bulmer effect depression and reached the highest values (Figure S9). We can confidently assume that selection was weak with β = 1, leaving available variance unused. A substantial part of the genotypic variance is inflated due to positive linkage disequilibrium, which means that the underlying genic variance is not that high. This is confirmed partially by Figure S8 on genic variance, notably for the extreme case of β = 1.

If He x Ho are penalized with β = 1, we could suppose that other relationships like Ho x Ho get promoted instead, and the fact that positive linkage disequilibrium is present would mean that genotypes concentrate either favorable homozygotes or unfavorable homozygotes. This situation, or when the genotypic variance is higher than the genic variance, is potentially favorable for selection to discriminate efficiently between favorable and unfavorable alleles, the extreme case being that of sublines or lineages with selection proceeding between them. This potentiality, however, did not materialize in better gains for β = 1, suggesting that there is a cause of inefficiency preventing selection from using that advantage. One such cause could be the fact of having unfavorable alleles segregating in the population with a risk of fixation, dragging gain downwards. The analysis on effect of selection and drift − that is, the accumulation of favorable allele and loss of unfavorable allele, or respectively the accumulation of unfavorable allele and loss of favorable alleles (Figure S10) − was not conclusive. As expected with negative correlation, they are equivalent, almost symmetrical, suggesting that selection has no advantages over drift, and whenever favorable alleles get fixed, unfavorable counterparts get fixed on the way.

# Supplementary Figures and Tables

## Supplementary Figures


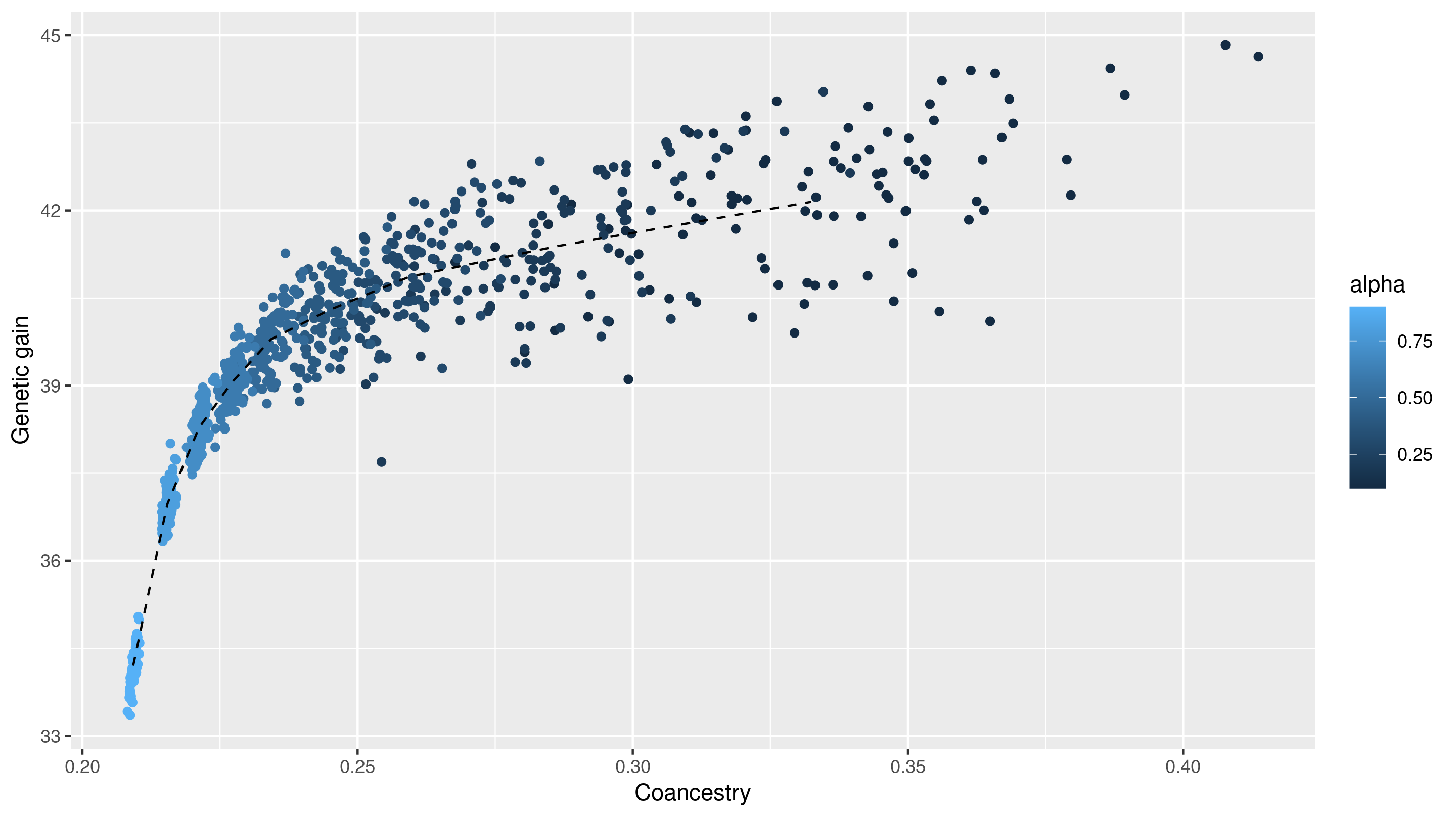


**Supplementary Figure 1.** The Pareto optimum graph : the x-axis is the coancestry of a population, and the y axis is the average genetic gain of a population. Each point is a simulation (one population), each color is a value of α (dark blue for α = 0.1, light blue for α = 0.9). The dotted line is the Pareto optimum predicted by OCS. Simulated with β = 0, and h^2^ = 0.5134.


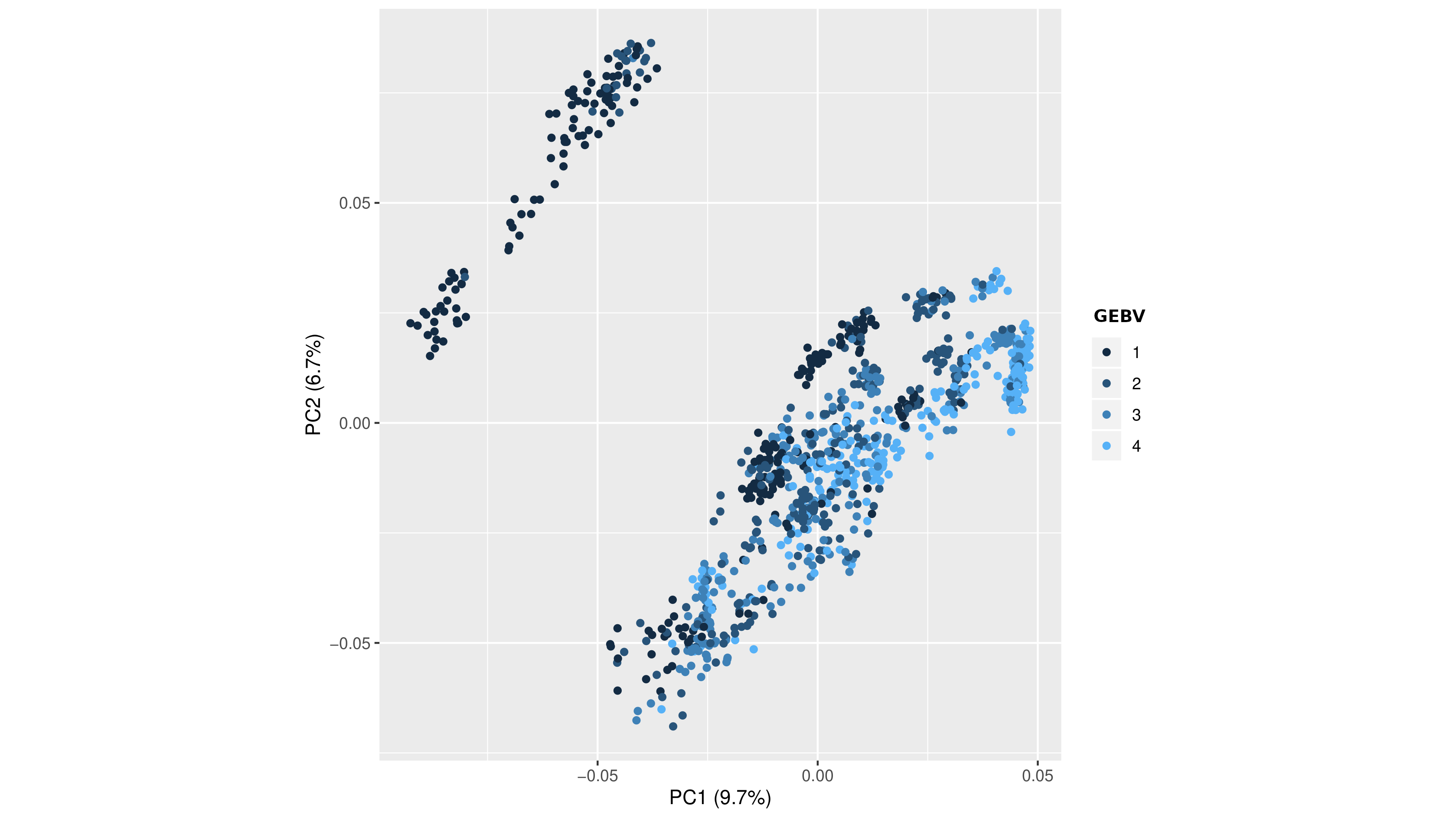


**Supplementary Figure 2.** Principal Component Analysis of the genotypes of the 1009 individuals of *Populus nigra*. Each point is an individual, and each color is the quartile of GEBVs (1 are the lowest GEBVs, 4 are the highest). The first two axes explained respectively 9.7% and 6.7% of the variance.


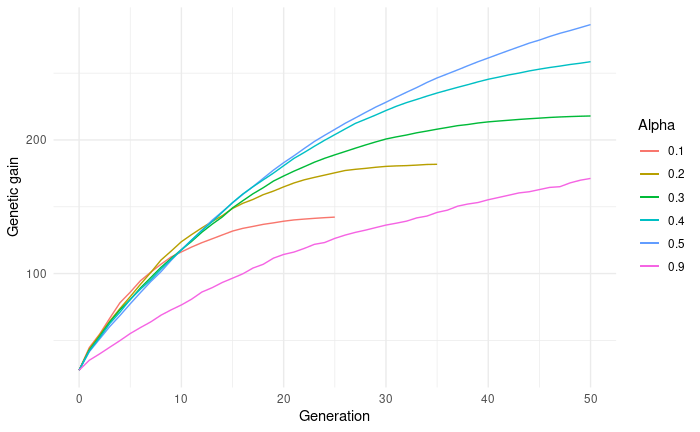


**Supplementary Figure 3.** Genetic gain over time (unshuffled dataset, β = 0, 1 replicate per α). The simulation stops when it reaches a plateau. Initial slope are higher for lower α, but they soon reach their selection plateau, making the intermediate and high values of α better in the long run. However, the horizon at which the highest values of α will outperform intermediate values of α is almost infinitely far. For such horizon, the framework should at least integrate mutations to make efficient predictions.


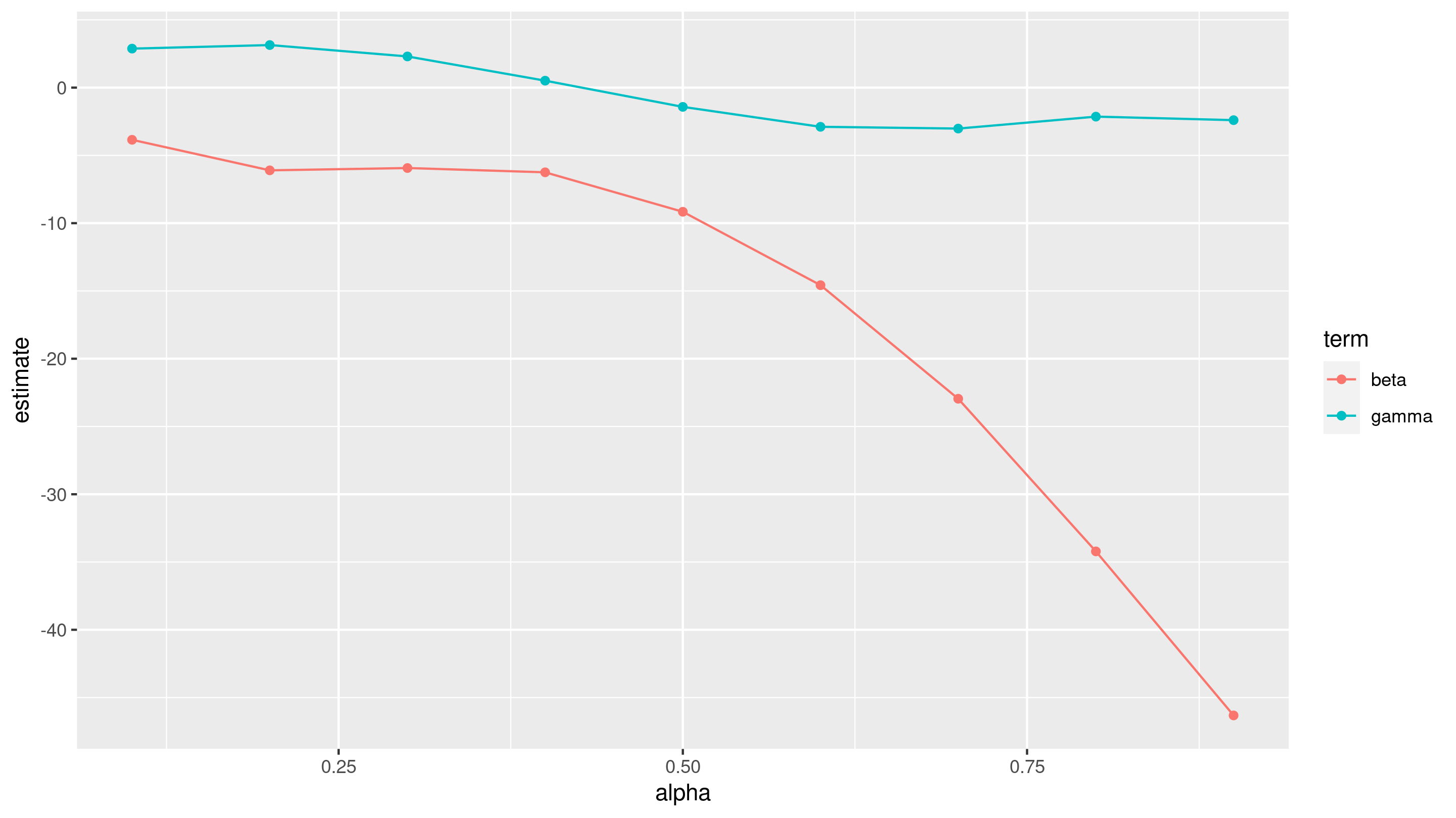


**Supplementary Figure 4.** Estimate of the effect of β and γ in the model (2), for genetic gain, for different values of α, at generation 20 and for h^2^ = 1.


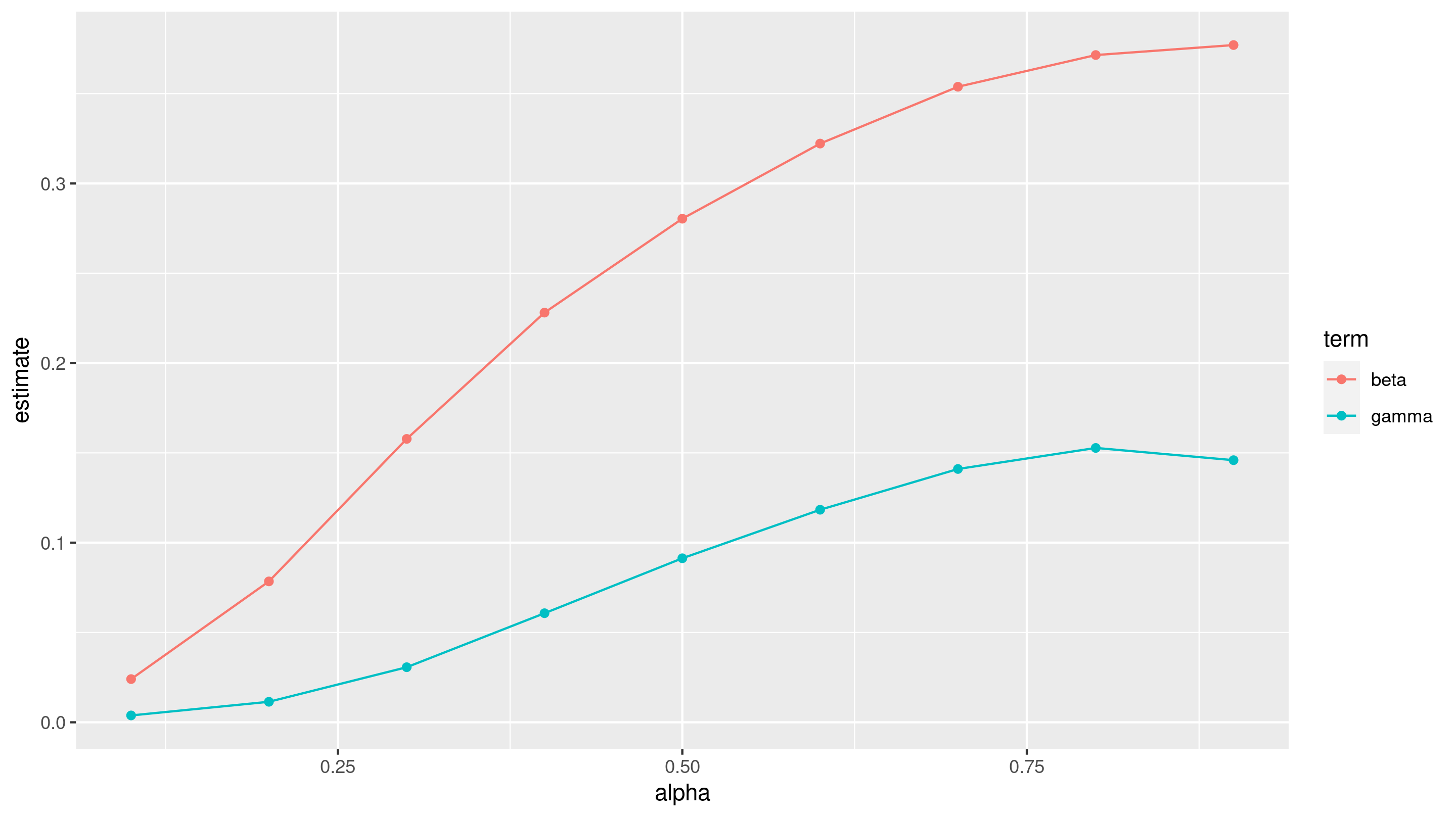


**Supplementary Figure 5.** Estimate of the effect of β and γ in the model (2), for coancestry, for different values of α, at generation 20 and for h^2^ = 1.


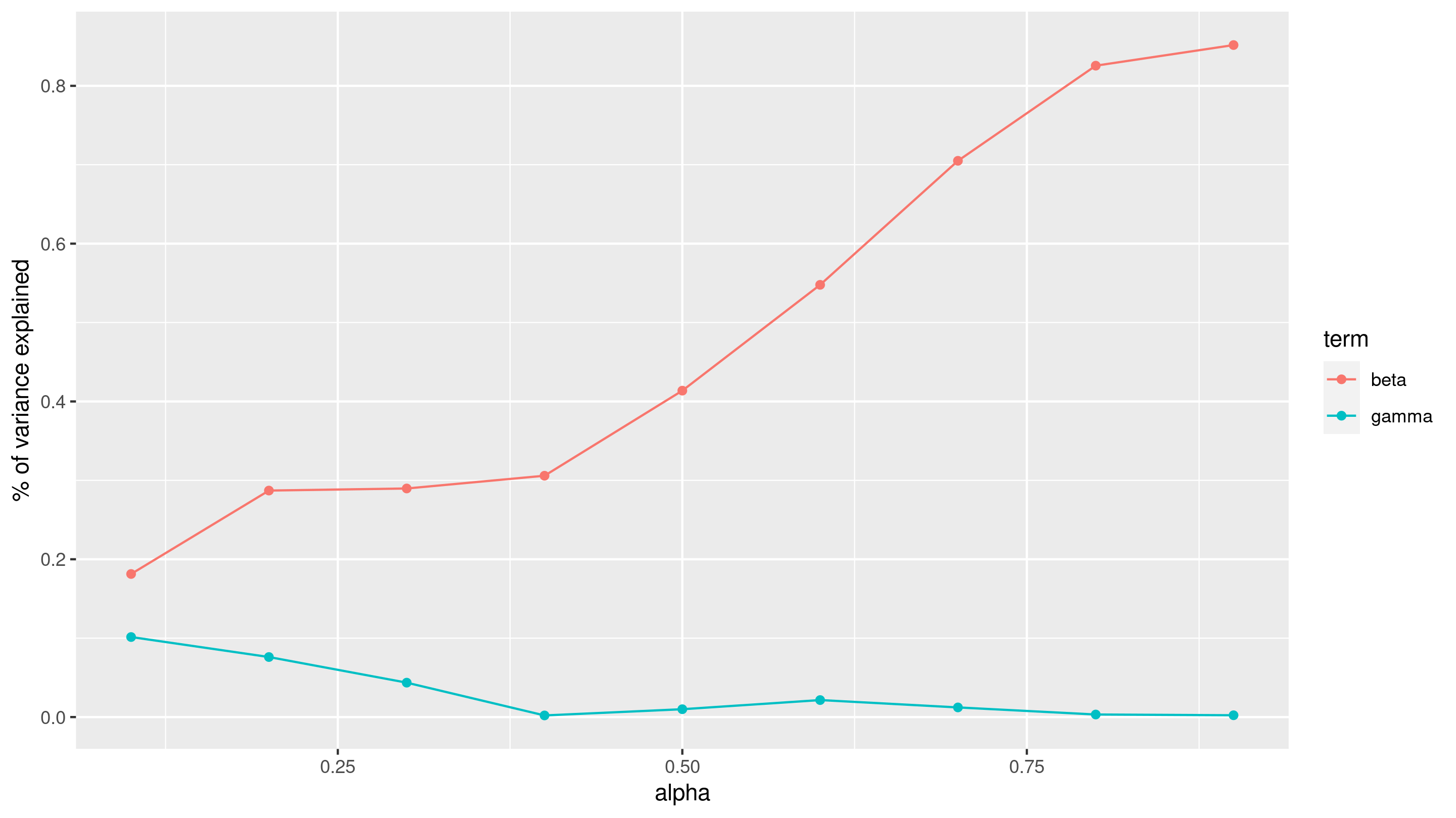


**Supplementary Figure 6.** Proportion of sum of squares of the effects of β and γ in the model (2), for genetic gain, for different values of α, at generation 20 and for h^2^ = 1.


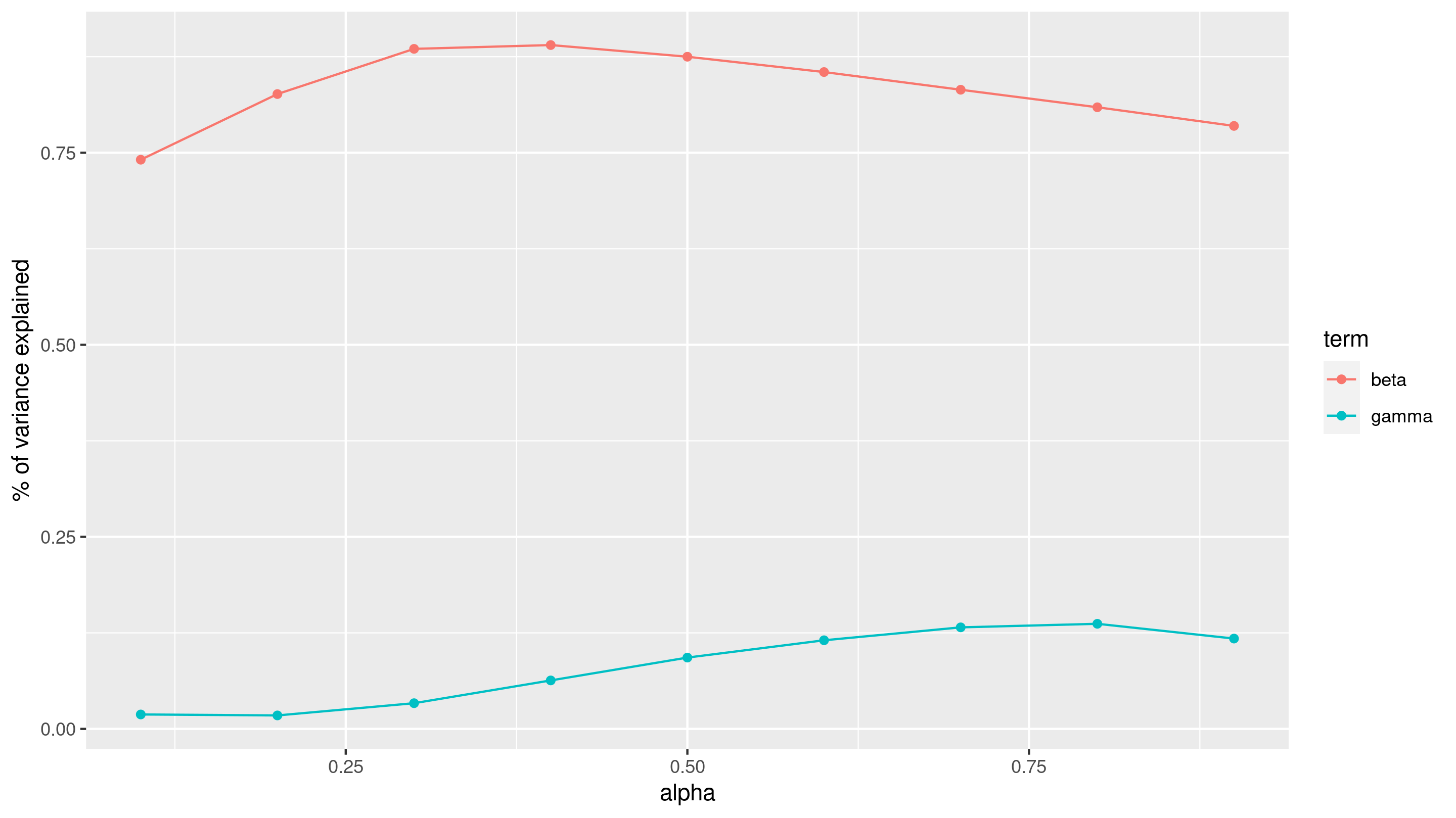


**Supplementary Figure 7.** Proportion of sum of squares of the effects of β and γ in the model (2), for coancestry, for different values of α, at generation 20 and for h^2^ = 1.


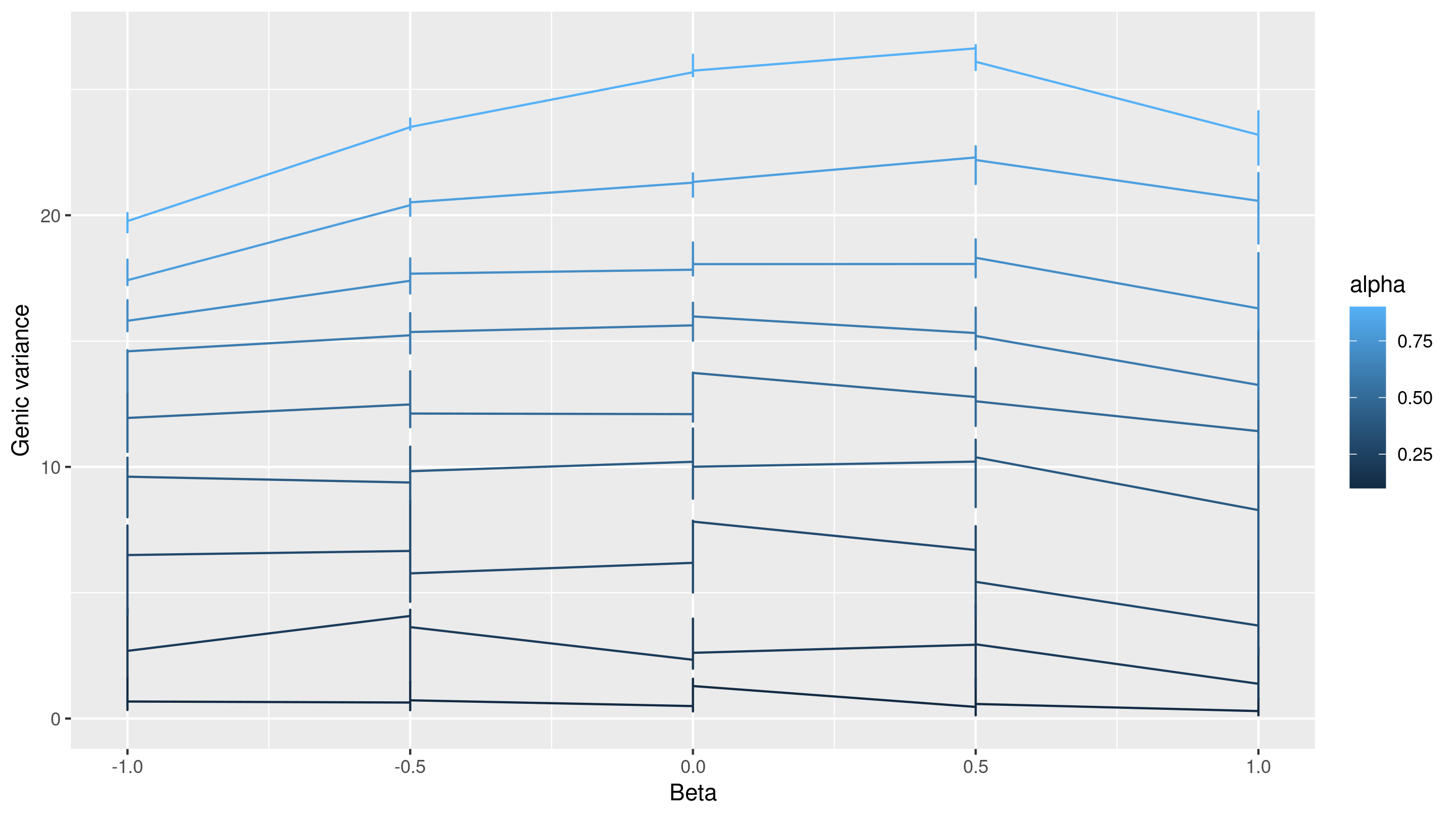


**Supplementary Figure 8.** Genic variance for different values of β and α (dark blue for α = 0.1, light blue for α = 0.9), at generation 20 and for h^2^ = 1.


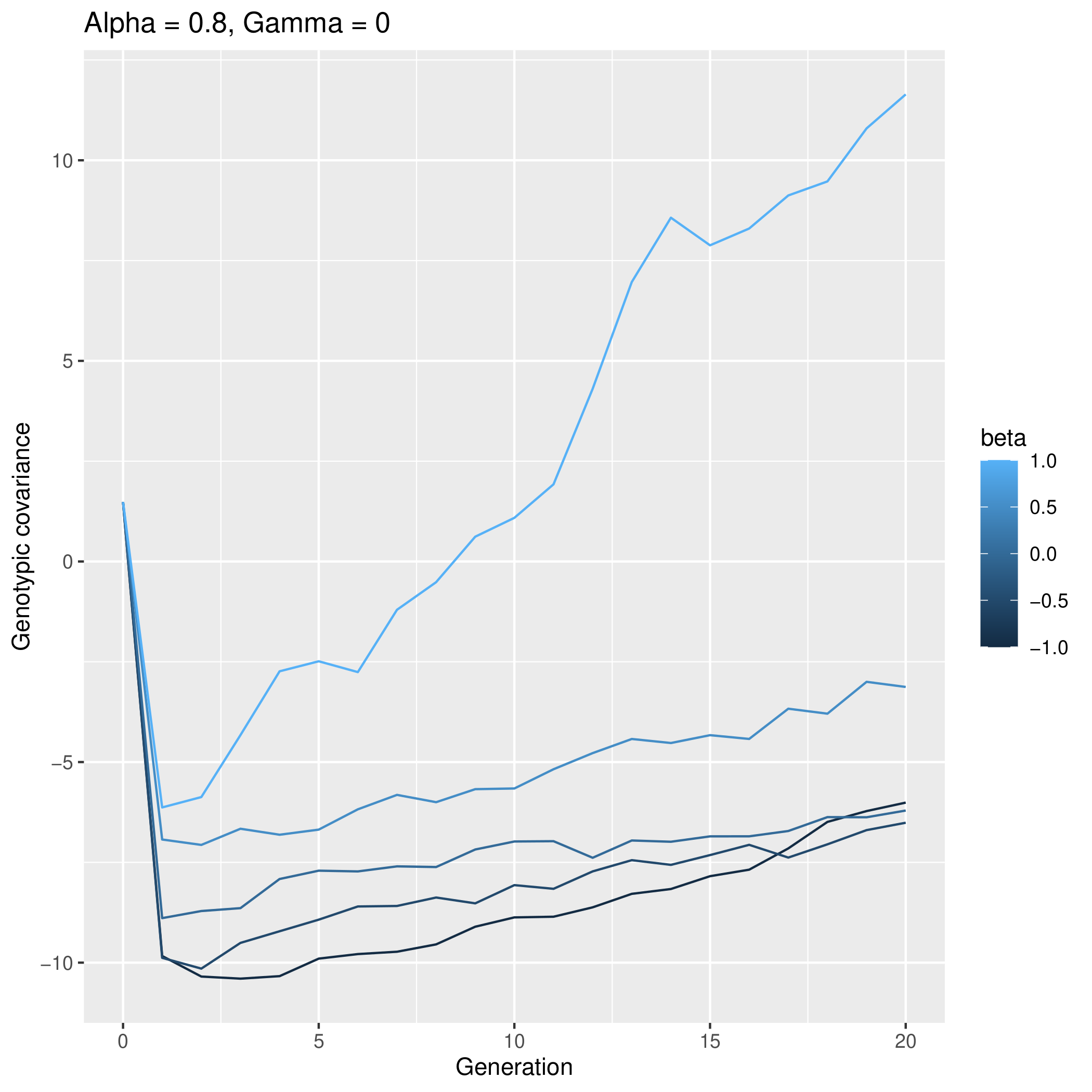


**Supplementary Figure 9.** Example of genotypic covariance for different values of β (dark blue for β = -1, light blue for β = 1), with α = 0.8, and h^2^ = 1.


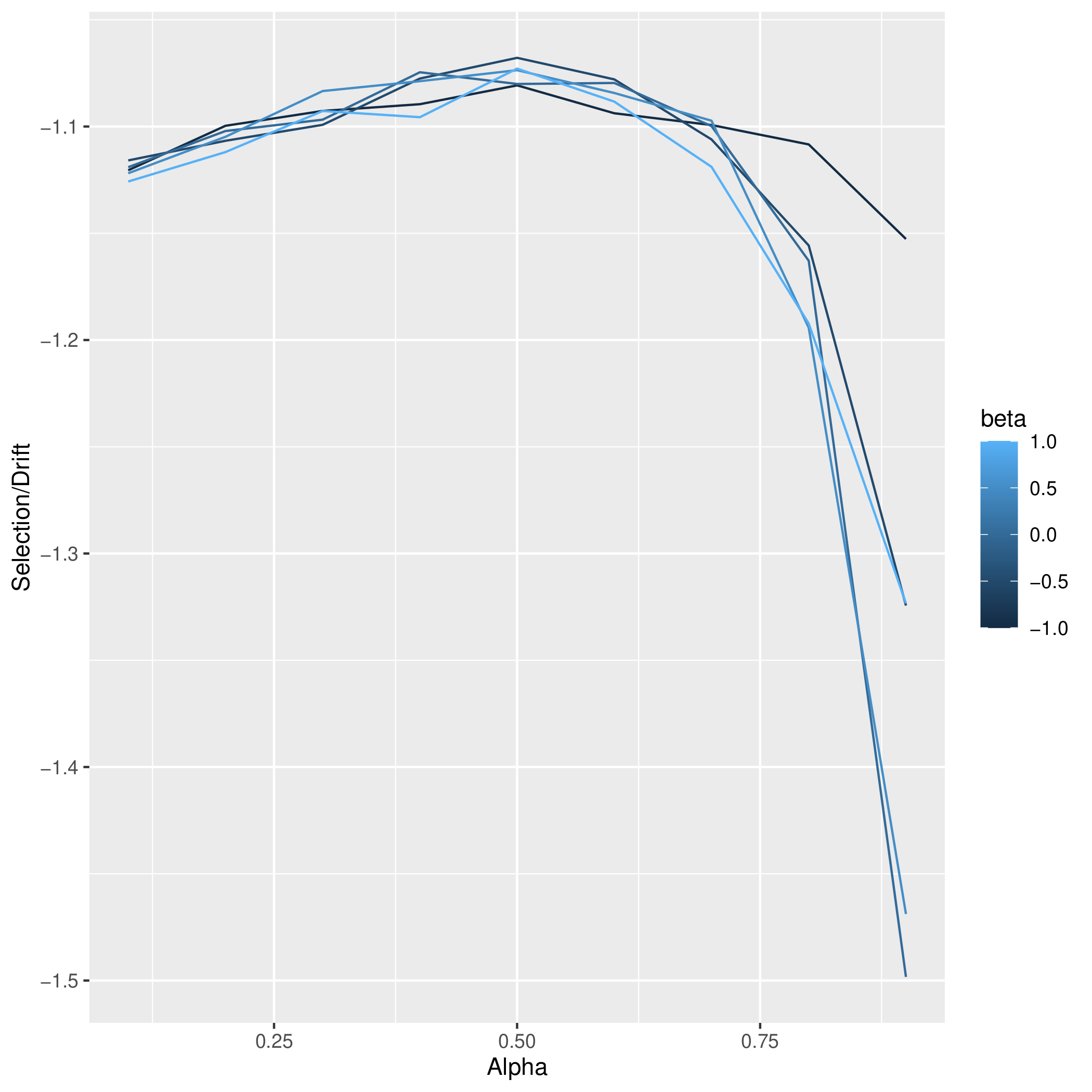


**Supplementary Figure 10.** Selection efficiency for different values of β (dark blue for β = -1, light blue for β = 1), at generation 20 and h^2^ = 1. Selection efficiency is the ratio of the accumulated allelic effects due to selection (fixed favorable allelic effect + lost unfavorable allelic effect) and the accumulated allelic effects due to drift (fixed unfavorable allelic effect + lost favorable allelic effect).
